# Syndecan-2 promotes perineural invasion and cooperates with K-ras to induce an invasive pancreatic cancer cell phenotype

**DOI:** 10.1186/1476-4598-11-19

**Published:** 2012-04-03

**Authors:** Tiago De Oliveira, Ivane Abiatari, Susanne Raulefs, Danguole Sauliunaite, Mert Erkan, Bo Kong, Helmut Friess, Christoph W Michalski, Jörg Kleeff

**Affiliations:** 1 Department of Surgery, Technische Universität München, Munich, Germany; 2 International Max Planck Research School, Martinsried-Munich, Germany

## Abstract

**Background:**

We have identified syndecan-2 as a protein potentially involved in perineural invasion of pancreatic adenocarcinoma (PDAC) cells.

**Methods:**

Syndecan-2 (SDC-2) expression was analyzed in human normal pancreas, chronic pancreatitis and PDAC tissues. Functional in vitro assays were carried out to determine its role in invasion, migration and signaling.

**Results:**

SDC-2 was expressed in the majority of the tested pancreatic cancer cell lines while it was upregulated in nerve-invasive PDAC cell clones. There were 2 distinct expression patterns of SDC-2 in PDAC tissue samples: SDC-2 positivity in the cancer cell cytoplasm and a peritumoral expression. Though SDC-2 silencing (using specific siRNA oligonucleotides) did not affect anchorage-dependent growth, it significantly reduced cell motility and invasiveness in the pancreatic cancer cell lines T3M4 and Su8686. On the transcriptional level, migration-and invasion-associated genes were down-regulated following SDC-2 RNAi. Furthermore, SDC-2 silencing reduced K-ras activity, phosphorylation of Src and - further downstream - phosphorylation of ERK2 while levels of the putative SDC-2 signal transducer p120GAP remained unaltered.

**Conclusion:**

SDC-2 is a novel (perineural) invasion-associated gene in PDAC which cooperates with K-ras to induce a more invasive phenotype.

## Introduction

Pancreatic ductal adenocarcinoma (PDAC) is still one of the most lethal human malignancies due to late clinical detection and its innate aggressiveness, stemming from its potential for early local invasion and metastasis [1-3]. Among a variety of genomic alterations in precursor lesions of PDAC (pancreatic intraepithelial neoplasia, PanIN), mutations in the gene encoding the small GTPase K-ras are most frequent and are currently believed to be one of the initiating steps in pancreatic carcinogenesis. Approximately 90% of the later stage pancreatic cancers are found to be K-ras mutated and seem to get "addicted" to K-ras overactivity (induced by K-ras mutation), which might in turn have implications for therapeutically targeting the K-ras pathway [4,5]. Apart from the mitogenic signal transduced by K-ras overactivity, it also increases the transdifferentiation from an epithelial cellular structure to a more mesenchymal phenotype. This particularly includes an increased migratory and invasive potential which is a "prerequisite" for invasion and distant metastasis. Regarding the local spread, perineural invasion (PNI) is a characteristic property of PDAC cells allowing the tumor to infiltrate adjacent organs and organ-structures. PNI is also believed to be one of the major causes of the severe pain syndrome accompanying advanced PDAC stages [6]. Within this process of invasion, tumor cells are interacting with and are producing (or are stimulating stromal cells to produce) proteins of the extracellular matrix (ECM), as well as cytokines, chemokines, cell-adhesion proteins and growth factors [7]. These active interactions then allow the tumor cells to coopt the tumor stroma [8]. In addition, cell surface receptors and other surface proteins such as proteoglycans are participating in tumor cell-stromal cell (i.e. nerves) communication. Among these, syndecans have recently attracted considerable attention. Syndecans are transmembrane heparan sulphate proteoglycans to which one or more glycosaminoglycan chains are covalently attached [9]. The heparan sulphate chains of syndecans have an extraordinary structural diversity, owing to differences in the position of the sulphate groups along the polysaccharide chain and epimerization of glucuronic acid residues to iduronic acid [10]. Four members of the syndecan family have been indentified to date, and all seem to be tissue-specific and to have distinct functions. We have previously characterized the expression of syndecan-1 in pancreatic, esophageal, gastric, colon, and liver human cancer tissues, and demonstrated that syndecan-1 is up-regulated in pancreatic cancer but not in the other gastrointestinal malignancies, revealing that its expression might play an important role in the pathobiology of this disease [11]. Recently, using a novel ex vivo perineural invasion model, we have identified 25 perineural-invasion-related genes [12]. Now, using the same ex vivo model, we identified syndecan-2 (SDC-2) expression to be up-regulated in pancreatic cancer cell clones which have an increased perineural invasive capacity. SDC-2 is a 21 kDa transmembrane protein with a short cytoplasmic domain that contains two constant regions (C1 and C2) separated by a variable region (V) that is unique for each syndecan family member. On immunoblots, SDC-2 is found as a stable dimer or oligomer at 40-50 KDa [13-16]. It is highly expressed in cells of mesodermal origin, and some reports have described it as a tumor suppressor [17], whereas others demonstrated that it functions as a promoter of tumorigenesis [18,19]. Recently, SDC-2 was associated with the migratory potential of melanoma cells and appears to act as a general protumorigenic receptor in those cells [20].

In our study, we report for the first time that SDC-2 promotes in vitro migration and invasion and that it cooperates with K-ras to induce a more malignant pancreatic cancer phenotype in vitro.

## Materials and methods

### Cell culture

Pancreatic cancer cells were routinely grown in DMEM or RPMI medium (Invitrogen, Karlsruhe, Germany), supplemented with 10% FBS (PAN Biotech, Germany), 100 U/ml penicillin and 100 μg/ml streptomycin, as described previously [21].

### Tissue sampling

Pancreatic tissue specimens were obtained from patients who underwent resection (median age 67 years, 32-85 years) for pancreatic cancer. Normal pancreas tissues were obtained through an organ donor program from previously healthy individuals. The Ethics Committee of the Universities of Heidelberg and Munich, Germany, approved the tissue collection. Written informed consent was obtained from all patients.

### Real-time quantitative polymerase chain reaction (QRT-PCR)

For SDC-2 mRNA expression analysis, 7 pancreatic cancer cell lines and 82 samples of human pancreas tissues (20 donors; 20 chronic pancreatitis; 42 PDAC), were used (clinical characteristics of PDAC patients: Table 1). Equipment and reagents were from Roche (Basel, Switzerland), except for the mRNA extraction kit (RNeasy Mini kit, Qiagen, Hilden, Germany) and the specific oligonucleotides (Metabion, Munich, Germany). A denaturing agarose gel was used to assess integrity of the extracted RNA. cDNA was prepared using the first-strand cDNA synthesis kit for RT-PCR according to the manufacturer's instructions (Fermentas International Inc., Canada). The primer sequence used for SDC-2 detection was 5'-ACATCTCCCCTTTGCTAACGGC-3' (forward) and 5'-TAACTCCATCTCCTTCCCCAGG-3' (reverse), Tm 58°C; human Cortactin (CTTN) 5'-TCACTTTGTAGGAAACTCATCTCCTT-3' (forward) and 5'-GTGTGCTACAGGAATTCAGATACAAT-3' (reverse), Tm 58°C; human WASF-1/2 5-TCCTGATGTTTTAAAAGAAGAAACACT-3' (forward) and 5-AAAAGTTTTTAACTCCTATAGGCAAGC-3' (reverse), Tm 58°C; human Cdc42 5-AGGCTCTCTAGTTTAATAAAAATCATGG-3' (forward) and 5- GTTTGTTTAATACATCTGAAAAGAATGC-3' (reverse), Tm 58°C, and carried out using the LightCycler™480 with the SYBR Green 1 Master (Roche). The relative expression was normalized to human (reference gene) GAPDH using the LightCycler™480 software release 1.5, version 1.05.0.39 (Roche).

**Table 1:** Characterization of PDAC tissues used in QRT-PCR analysis

**Total Patients**	42
***Median Age***	66
***Sex***	
***Males***	18
***Females***	24
***T1***	00
***T2***	03
***T3***	39
***N0***	09
***N1***	33
***M0***	39
***M1***	03
***G1***	02
***G2***	23
***G3***	17

### Immunohistochemistry

Immunohistochemistry was performed as previously described [12,22] using 92 formalin-fixed, paraffin-embedded samples (clinical characteristics: Table 2). Rabbit anti-human pancytokeratin antibody (Abcam) and rabbit anti-human syndecan-2 antibody (Invitrogen) were used at a concentration of 1:100, diluted in antibody diluent (DAKO, Glostrup, Denmark). To confirm the specificity of the primary antibody, tissue sections were incubated with negative control rabbit IgG or the syndecan-2 immunizing peptide (R&D Systems, MN, 1:10 dilution). Semi-quantitative evaluation was performed by 2 independent researches as follows: SDC-2 tumor cell expression versus no tumor cell expression and/or SDC-2 peritumoral expression versus no peritumoral expression.

**Table 2:** Characterization of PDAC tissues used in IHC analysis

**Total Patients**	92
***Median Age***	67
***Sex***	
***Males***	57
***Females***	35
***T1***	01
***T2***	04
***T3***	87
***N0***	13
***N1***	79
***M0***	76
***M1***	16
***G1***	03
***G2***	42
***G3***	47

### Immunoblot analysis

Immunoblot analysis was performed as previously described [23], using the following antibodies: rabbit anti-syndecan-2 (Invitrogen), rabbit anti-phospho-ERK, mouse anti-total-Src (7 G9), mouse anti-phospho-Src (Tyr416; all from Cell Signaling, Danvers, MA), rabbit anti-ERK-2 (Abcam, Cambridge, UK), mouse anti-p120GAP and rabbit anti-GAPDH (both Santa Cruz Biotech., Santa Cruz, CA). For the analysis of serum-free cell supernatants (40 μl), these were subjected to SDS-PAGE under reducing conditions, without pre-heating (data not shown). To identify the SDC-2 core protein (21 KDa), we enzymatically treated Su8686 cleared cell lysates with 6 M urea in sodium acetate (ph 4.5), and boiled them at 95°C for 10 min. Subsequently, the samples were centrifuged at 12.000 rpm (+4°C), for 10 minutes and the supernatants used for further analyses. Additionally, the samples were treated twice with heparinase III, 1 mU/ml or 2 mU/ml, at 42°C for 30 min. Densitometric analyses were performed using the "Image J" software http://rsbweb.nih.gov/ij/.

### RNA interference

For transient mRNA silencing, approximately 15 × 10^4 ^cancer cells per well in 6-well plates were transfected with 20 μM RNAi/well syndecan-2 Silencer Selected™ siRNA (Ambion/Applied Biosystems, Carlsbad, CA), sequence s12637, 5'-GCUUCAGGAGUGUAUUCCUAtt-3' (sense), 5'-UAGGAUACACUCCUGAAGCtt-3' (antisense) and sequence s12635, 5'-UGACCUUGGAGAACGCAAAtt-3' (sense), 5'-UUUGCGUUCUCCAAGGUCAta-3' (antisense) using Hiperfect™ transfection reagent (Qiagen, Hilden, Germany). Scrambled siRNA (Ambion) was used as a control.

### Matrigel invasion assay

Invasion assays were performed as previously described [24,25]. 15 × 10^3 ^T3M4 and Su8686 cells which had been transfected with SDC-2 or control siRNA (scrambled) prior to seeding into the Boyden chamber were seeded into the top chamber and were incubated for 24 h. Cells adhering to the lower surface were fixed with 75% methanol and 25% acetone and were stained with Mayer's Hematoxylin. The invading cells were counted under a light microscopy (Zeiss Axioskop 40). The assays were performed in triplicate and were repeated three times.

### Proliferation assays

Anchorage-dependent cell growth was determined using the 3-(4,5-methylthiazol-2-yl)-2,5-diphenyltetrazolium bromide (MTT) colorimetric growth assay. Briefly, 2,000 cells/well were plated in 96-well plates and were cultured for up to 3 days. Each day, cell growth was determined by adding MTT solution (50 μg/well) for 4 h. Cellular MTT was solubilised with acidic isopropanol and optical density was measured at 570 nm. The doubling time was calculated for the exponential growth phase. All experiments were performed 3 times in triplicates.

### Immunofluorescence analysis

Cells were seeded at 1 × 10^5^/well, in 8-wells glass chamber slides (NUNC, New York, NY) and were grown overnight. The cells were then fixed with 4% Paraformaldehyde, washed with 1 × PBS, and permeabilized with 0.1% Triton X-100/PBS, followed by blocking with 20 mM glycine/PBS. Rabbit anti-human syndecan-2 was added at a dilution of 1:100 and was incubated overnight at 4°C, followed by incubation with anti-rabbit FITC-conjugated antibody (dilution of 1:1000) and the DAPI dye (dilution of 1:20000, both from G&E Health Care, Backinghamshire, UK). Images were taken on a Zeiss Axioskop 40 or Apotome™(both Carl Zeiss, Jena, Germany).

### Migration assay

T3M4 and Su8686 cells were seeded into μ-dish-35 mm low culture inserts according to the manufacturer's instructions (ibidi Integrated BioDiagnostics, Munich, Germany). After 12 hours, the silicon brackets were removed and cell migration was observed in 12-hour intervals, until 48 hours post RNAi. Images were taken using a Canon A610 camera (Canon Inc., Tokio, Japan) on an Axiovert 40 CFL inverted microscope (Carl Zeiss, Jena, Germany). Analyses were carried out using the Adobe Photoshop™ CS4 software (Adobe Systems Inc., San Jose, CA) and GraphPad Prism ™ 5 (GraphPad Software, Inc., La Jolla, CA).

### Immunoprecipitation assays

For SDC-2 immunoprecipitation (IP), a protein G-agarose kit was used (IP50, Sigma-Aldrich, St. Louis, MO), according to the manufacturer's instructions. Pancreatic cancer cells (Su8686 and BxPC3) were lysed on ice in 1 × IP buffer and were centrifuged for 10 minutes at 12.000 rpm. Nuclei and debris were removed and the samples were transferred into IP columns for incubation with rabbit anti-human SDC-2 antibody. After 2 hours +4°C, the eludate was incubated with protein G-agarose at +4°C, overnight. Precipitates were washed 5 times with ice-cold 1× IP buffer, eluted with 1× Laemmli sample buffer, and boiled for 10 minutes. Bound proteins were subjected to SDS-polyacrylamide gel electrophoresis and were electrotransferred onto nitrocellulose membranes. The membranes were then subjected to mouse p120GAP antibody (Santa Cruz Biotech.) or rat anti-human SDC-2 (R&D Systems, Minneapolis, MN).

### Ras activity assay

Fresh cell lysates were treated according to the manufacturer's instructions (Millipore/Upstate, Temecula, CA). Briefly, total cell lysates, cleared of nuclei and debris by 5 min centrifugation, were incubated for 90 min, at 4°C, with beads coated with a fusion protein (GST-Raf1-RBD) consisting of GST fused to the Ras binding domain of Raf-1. Beads were washed three times with ice cold 1× MLB buffer. The bound protein was eluted by 10 min boiling with 2× Laemmli sample buffer and was analyzed by immunoblotting for Ras activity.

### Statistical analysis

Differences in survival between the tumor cell expression/no-expression and expression around tumor cells/no expression groups were analyzed using the Kaplan-Meier method. Results are expressed as mean ± standard error of the mean (SEM), unless otherwise stated. Significance was set at p < 0.05. The statistical analyses were performed using the GraphPad Prism™ 5 software (GraphPad Software).

## Results

Using an ex vivo model of perineural invasion in pancreatic cancer [12], we have found upregulation of syndecan-2 in nerve-invasive pancreatic cancer cell clones. In this study, we used descriptive methods on human pancreatic cancer tissues and genetic manipulation in vitro to further assess the role of syndecan-2 in pancreatic cancer with an emphasis on elucidating its role in invasion and motility as surrogates of the locally advanced and metastatic disease.

### SDC-2 is upregulated in nerve-invasive pancreatic cancer cells

**Figure 1:**
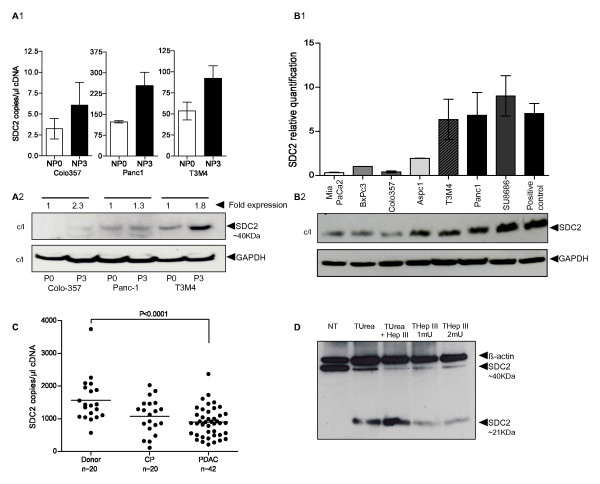
**(A-D) A-1, QRT-PCR, SDC-2 mRNA levels in nerve invasive cancer cells; A-2, SDC-2 protein levels in cell lysates (c/l); GAPDH as a loading control; "Fold expression" has been calculated using densitometry of the immunoblots which were normalized to GAPDH**. **B-1**, QRT-PCR, SDC-2 mRNA levels in PDAC cell lines (normal colon as a positive control). **B-2**, SDC-2 protein levels in PDAC cell lysates (normal colon as positive control). **C**, QRT-PCR, SDC-2 mRNA levels in human normal pancreas (donor), CP and PDAC tissues. **D**, enzymatically treated SU8686 cell lysates; NT = not treated; (1) = 6 M urea in sodium acetate ph 4,5 treated; (2) = 6 M urea in sodium acetate ph 4,5 plus 1 mU/ml Heparinase III; (3) = 1 mU/ml Heparinase III treatment; and (3) = 2 mM/ml Heparinase III treatment.

**Figure 2:**
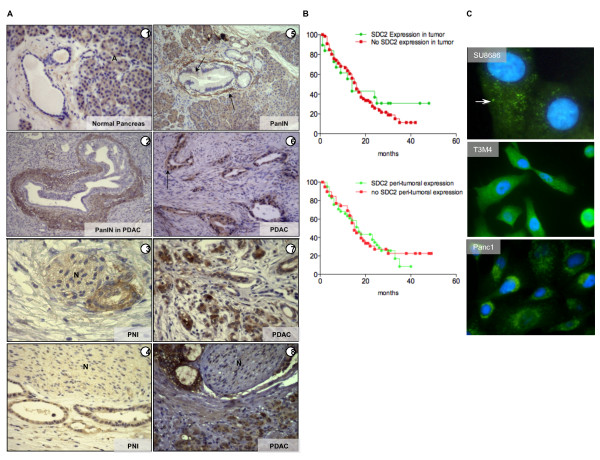
**(A-C) A, IHC staining of different pancreatic tissues using an anti-SDC-2 antibody (A1-7) and an anti-pan-cytokeratin antibody (A8); A: acinar cells, N: nerve(s)**. **B**, Kaplan-Maier survival curves of patients with tumor cell-expressing/non-expressing tissues or with/without expression around tumor cells; **C**, immunofluorescence staining of Su8686, T3M4 and Panc-1 pancreatic cancer cells using an anti-SDC-2 antibody.

We compared expression of SDC-2 in less nerve-invasive pancreatic cancer cell clones (passage 0, i.e. before ex vivo nerve invasion) to clones which had been subjected to ex vivo nerve invasion (passage 3) using quantitative RT-PCR (QRT-PCR) and immunoblot analysis. These assays revealed an upregulation of SDC-2 mRNA and protein (cell lysates, c/l) levels in passage 3 nerve-invasive cancer cell clones (Figure 1, 2); however, this was not constantly reflected in the secretory compartment where - due to unknown reasons such as differential secretion or cleavage - upregulation of SDC-2 was only observed in Colo-357 passage 3 cells (data not shown). RT-PCR of mRNAs from a larger set of pancreatic cancer cell lines substantiated these results by demonstrating that in seven cell lines, SDC-2 transcripts were found (Figure 1, B1, normal colon = positive control). The cell lines, in which SDC-2 was transcribed, were also SDC-2-positive on the protein level (seven tested cell lines, normal colon as a positive control, Figure 1, B2). Interestingly, and in line with previous studies, the SDC-2 core protein was found in its dimerized form at 42 kDa (Figure 1, D). Because di- and oligomerization are essential for the function of syndecans [26,27] and are dependent on the presence of glycosaminoglycan side chains [9], we decided to enzymatically deglycate the cell lysates. Using this assay, we identified the unglycanated form of SDC-2 (at 21 kDa) in immunoblot analysis (Figure 1, D. Su8686 cells). To gain insight into the potential function of SDC-2 in vivo, we quantitatively analyzed its mRNA expression in bulk pancreatic tissues from healthy donors (normal pancreas, NP, n = 20), from chronic pancreatitis patients (CP, n = 20) and pancreatic ductal adenocarcinoma (PDAC, Table 1. n = 42). Unexpectedly, SDC-2 mRNA was downregulated in PDAC (p < 0.001, Figure 1, C).

### SDC-2 expression in pancreatic tissue

In a next step, we performed immunohistochemistry on tissue samples of normal pancreas, pancreatic intraepithelial neoplasia (PanIN), pancreatic cancer (PDAC), and pancreatic cancer with perineural invasion (PNI), (Figure 2A, Table 2). While in the normal pancreas, there was a moderate to strong staining for SDC-2 in acinar cells and no or weak staining around ducts, there was strong immunoreactivity for SDC-2 around pancreatic intraepithelial neoplasia lesions (Figure 2, A2, 5; both in PanINs from malignant and non-transformed tissues, arrows). In PDAC, different patterns of cellular/stromal distribution of SDC-2 were found: around some cancer structures, SDC-2 deposition was seen at different intensities (Figure 2, A6, 7) whereas some cancers showed only cytoplasmic cancer cell positivity for SDC-2 (Figure 2, A6; arrow); in other PDACs, nuclear SDC-2 staining was present (Figure 2, A7). Cancer cells which invaded pancreatic nerve structures were generally SDC-2-positive. Using a semi-quantitative evaluation, we compared the SDC-2 expression patterns in tissues from 92 pancreatic cancer patients. While in 48 tissues, peritumoral SDC-2 expression was found (out of these, a minority also showed cytoplasmatic cancer cell staining), in 24 tissues there was SDC-2 positivity in the cytoplasm of the cancer cells. 20 tissue samples were negative for SDC-2. Kaplan-Meier survival analysis of the groups with versus without SDC-2 cancer cell expression and peritumoral versus no peritumoral SDC-2 expression demonstrated that none of the observed expression patterns were associated with survival (Figure 2B; upper panel, median survival 14 vs. 16 months; lower panel, median survival 16 vs. 15 months). According to these results, we hypothesized that these expression patterns (and secretion patterns) might be reflected in vitro; however, immunofluorescence analyses only confirmed a granular cytoplasmic localization of SDC-2 (Figure 2C; comparable to SDC-2 localization in prostate cancer cells [28]). Thus, in a next step, we analyzed the function of SDC-2 in pancreatic cancer cells in vitro.

### SDC-2 knockdown decreases pancreatic cancer cell migration and invasion and reduces K-ras/MAPK pathway signaling

**Figure 3:**
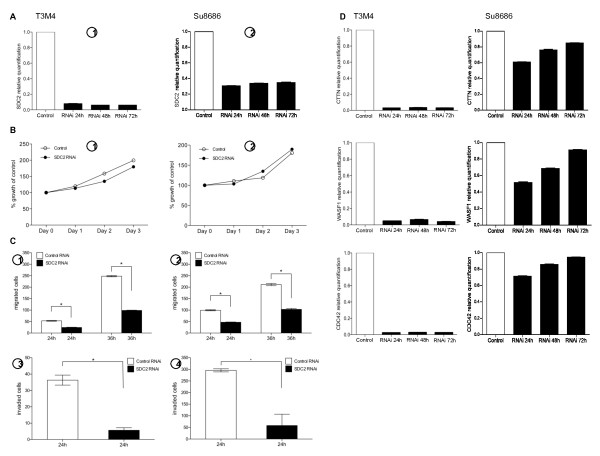
**A-D, functional assays following silencing of SDC-2 in T3M4 and Su8686 cells**. **A1-2**, QRT-PCR following SDC-2 RNAi; **B1-2**, proliferation assays; **C1-2**, migration assays; **C3-4**, matrigel invasion assays; **D**, QRT-PCR analysis of the expression levels of migration-and cytoskeleton-associated gens 24, 48 and 72 hours after SDC2 silencing.

**Figure 4:**
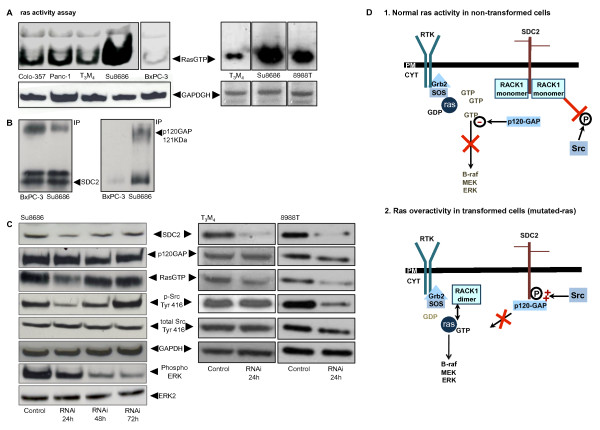
**A, ras-activity assay in pancreatic cancer cell lines Colo-357, Panc-1, T3M4, Su8686, 8988 T (all K-ras-mutated) and BxPC3 (wild-type K-ras); **B**, immunoprecipitation with an anti-SDC-2 antibody; lysates from BxPC3 and Su8686 cells (left panel); blotting with an anti-p120GAP antibody (right panel); **C**, immunoblot following SDC-2 RNAi: p120GAP, ras-activity (ras-GTP), total and phospho-Src (Tyr416), total and phospho-ERK; "Fold expression" has been calculated using densitometry of the immunoblots normalized to GAPDH; **D**, schematic SDC2 signaling in **1**, non-transformed cells (WT ras) with "normal" ras activity and in **2**, (malignant) transformed cells (ras-mutated) with ras overactivity**.

Because SDC-2 has been associated with tumorigenicity in sarcoma, fibrosarcoma, melanoma and colon cancer cells [20,29-31], we set out to determine whether modulation of SDC-2 expression in pancreatic cancer cells would affect their motility and their invasive capacity in vitro. To this end, we used the T3M4 (previously used for the PNI experiments, [12]) and the Su8686 cell lines due to their SDC-2 expression levels and K-ras mutational status [32]. Using a specific siRNA, SDC-2 levels were significantly reduced in both cell lines for up to 72 h (Figure 3, 1, 2; for reduction of SDC-2 protein levels following RNAi see Figure 4C). Since syndecans have been suggested to modulate proliferation signaling via receptor tyrosine kinase interference [27,33-37], we then tested the effects of SDC-2 RNAi on cell growth using standardized MTT assays; however, no significant differences were observed comparing control RNAi-transfected to SDC-2 RNAi-transfected cells (Figure 3, 1, 2). In contrast, cell migration was significantly reduced 24 h and 36 h after RNAi (Figure 3, 1, 2; p < 0.0001 at 24 h and 36 h for both cell lines, respectively), as was the number of invaded cells in the matrigel invasion assay (Figure 3, 4; p = 0.001 at 24 h and 36 h for both cell lines, respectively). To corroborate these findings on a transcriptional level, we assessed expression levels of three migration- and cytoskeletal organization-associated genes: cortactin (CTTN) has been shown to regulate interactions between components of adherens junctions, to be involved in cytoskeleton organization, cell adhesion and to be a substrate for Src [38]; Wiskott-Aldrich syndrome protein family member 1 (WASF1) is involved in transduction of signals from receptors on the cell surface to the actin cytoskeleton. Recent studies have demonstrated that these protein family, directly or indirectly, associate with the small GTPase CDC42 (cell division cycle 42), known to regulate formation of actin filaments, and the cytoskeletal organizing complex, Arp2/3 [14,39]. Compatible with our functional observation that SDC-2 regulates cell migration and invasiveness, expression levels of these genes were strikingly reduced 24 h after SDC2 RNAi in T3M4 cells (and remained suppressed for up to 72 h; Figure 3D). In Su8686 cells, SDC-2 RNAi induced an approximately 40% reduction in CTTN and WASF1 levels and a 25% reduction in CDC42 expression after 24 h; after 72 h expression levels returned to baseline (Figure 3D, right panel).

Recent reports have shown that syndecan-2 interferes with K-ras/MAPK signaling via association with p120-GAP to function as an active switch signal for Src in cells transformed with oncogenic ras [40-42]. Since the Su8686 cell line, which we used in our experiments, is K-ras-mutated and has thus constitutive overactivity of K-ras signaling (higher than the other tested cell lines; Figure 4A, upper panel), we determined whether SDC-2 silencing would affect K-ras and MAPK activity. In a first step, we immunoprecipitated SDC-2 in BxPC3 (low K-ras activity, wildytpe K-ras [43,44]), and Su8686 cells (high K-ras activity, K-ras-mutated [32]) and analyzed whether p120-GAP was bound to the precipitated SDC-2: these experiments demonstrated that in BxPC3 cells, no p120-GAP was bound to SDC-2 (Figure 4B, right) whereas in Su8686 cells, a strong p120-GAP signal was detected (Figure 4B, right). We thus concluded that p120-GAP binding to SDC-2 was associated with the K-ras activity of the respective cell lines. To determine whether there was a direct relation between SDC-2 expression levels and K-ras/MAPK, p120 and Src signaling, we silenced SDC-2 in Su8686, T3M4 and 8988 T pancreatic cancer cells and analyzed ras activity, levels of p120-GAP and phosphorylation of Src (Tyr416) and of ERK (Figure 4C). While p120-GAP was not reduced, ras activity and phospho-Src levels were significantly lower in Su8686 and 8988 T cells (both cell lines demonstrated the comparably highest Ras activity, Figure 4A) at 24 h after RNAi. Levels of phosphorylated ERK decreased gradually, reaching the minimum at 72 h in SDC-2 transfected Su8686 cells. These results suggest that SDC-2 signaling merges into the K-ras/MAPK pathway. Interestingly, no modifications of these proteins were detected in T3M4 and Panc1 cancer cells (Figure 4C and data not shown).

## Discussion

Local and perineural invasion are prominent characteristics of pancreatic cancer which are thought to contribute to its particular aggressiveness and also to the severe pain syndrome associated with the disease [22,45]. A deeper understanding of the underlying molecular mechanisms is strongly needed to be able to directly target these properties. We have recently developed a novel in vitro model of perineural invasion of pancreatic cancer cells which we used to determine a number of genes which are deregulated when the cells become more invasive [12]. Using this model, we found syndecan-2 upregulation in nerve-invasive pancreatic cancer cell lines. Most importantly, we found that syndecan-2 modulates invasiveness of pancreatic cancer cells in culture and may interferes with K-ras/MAPK signaling as one of the predominantly deregulated pathways in PDAC. This is of particular interest since it has been demonstrated by a number of reports that there is a link between syndecan-2 and oncogenic ras signaling. Firstly, in non-transformed-ras cells, the scaffolding protein RACK1 (receptor for activated C-kinase 1) is bound as a heterodimer to the cytoplasmic domain of SDC-2. Furthermore, it has been demonstrated that in cells transformed by oncogenic ras, RACK1 is no longer bound to the SDC-2 cytoplasmatic tail, and that the GTPase activation protein GAP (p120-GAP) that negatively regulates ras activity, and normally is not translocated to the plasma membrane was highly expressed and that its localization overlapped with SDC-2. This complex (SDC2/p120-GAP) can provide a docking site for Src to prosecute tyrosine kinase activity, maintaining Src in an active form [40]. In contrast, following oncogenic transformation by overactivation of ras, RACK-1 is dimerized and thus no longer bound to the SDC-2 tail. This leads to a) a perpetuation of the K-ras signal by the RACK1 dimer and b) to binding of p120-GAP to SDC2 and thus to the loss of the inhibitory p120-GAP signal on K-ras-GTP [41,42]. It is important to point out that when we silenced SDC-2 expression, p120GAP protein levels were not altered, suggesting that p120GAP might negatively regulate ras activity. These results would be in line with recent reports and underline the importance of syndecan-2 signaling in K-ras overactive (transformed) cells (Figure 4D, 1: normal ras activity; 2: ras overactivity). Furthermore, we have silenced SDC2 in T3M4 and Panc1 pancreatic cancer cell lines. Here, we did not find the changes as observed in Su8686 and 8988 T cells, suggesting that these effects are due to the different Kras-status of these cells (although Panc1 and T3M4 cells also have a high SDC-2 expression (Figure 1B), K-ras is not constantly active). Interestingly, a recent study showed that there are two classes of pancreatic cancer cell lines: those which require K-ras to maintain viability (K-ras dependent) and those which do not (independent) [4]. Our findings that silencing of SDC-2 not only reduces Src Tyr416 phosphorylation and ras activity, but also (downstream) ERK phosphorylation, might be interpreted in two ways: firstly, Su8686 pancreatic cancer cells are K-ras-independent and syndecan-2 RNAi signals to K-ras as well as directly into the MAPK pathway or secondly, Su8686 cells are K-ras-dependent and ablation of the (intact) K-ras signal effects its downstream targets and thus reduces invasiveness and migration. Our in vitro results using pancreatic cancer cells are similar to a previously published report [20], where SDC-2 induced the migratory activity of melanoma cells through activation of FAK, which directly and indirectly interacts not only with MAPK and Src signaling, but also with CDC42, Cortactin and WAVE.

Furthermore, a recent study from Hrabar et al. [46] showed comparable immunohistochemical results regarding SDC-2 expression in pancreatic cancer. In contrast to our results, SDC-2 expression correlated with survival on multivariable analysis, and patients with higher SDC-2 expression had a distinctly longer survival. Differences in the number of patients analyzed may explain these different results.

The exact mechanisms underlying these differences and our functional observations will be the subject of further analyses; however, reduction of the invasive and migratory potential without effecting proliferation of the cancer cells could be an appealing approach to target a de-regulated system without inducing too much selective pressure on the cancer cells which might result in tumor-therapy evasion.

## Conclusions

In conclusion, we demonstrate that syndecan-2 is an important mediator of pancreatic cancer cell invasiveness and that it cooperates with oncogenic K-ras in the induction of a (more) malignant phenotype. The results of this study thus lay the basis for an in-depth analysis of the interference in de-regulated syndecan signaling in pancreatic cancer (perineural) invasion.

## Abbreviations

PDAC: Pancreatic ductal adenocarcinoma; CP: Chronic pancreatitis; NP: Normal pancreas; PNI: Perineural invasion; SDC-2: Syndecan-2; RNAi: RNA interference; IHC: Immunohistochemistry; IF: Immunofluorescence; WB: Western blot; WT: Wild-type; IP: Immunoprecipitation.

## Competing interests

The authors declare that they have no competing interests.

## Authors' contributions

TDO, IA, SR and DS carried out the experiments; TDO, IA, SR, DS, ME, BK and CWM analyzed data. TDO, CWM, IA, HF and JK drafted the manuscript. CWM, TDO, ME, BK, HF and JK designed the study. HF, JK and CWM coordinated the study. All authors read and approved the final draft of the manuscript. TDO and IA contributed equally to the study.
